# Stomatal penetration: the cornerstone of plant resistance to the fungal pathogen *Zymoseptoria tritici*

**DOI:** 10.1186/s12870-024-05426-5

**Published:** 2024-08-02

**Authors:** Mélissa Battache, Marta Suarez-Fernandez, Madison Van’t Klooster, Florence Cambon, Andrea Sánchez-Vallet, Marc-Henri Lebrun, Thierry Langin, Cyrille Saintenac

**Affiliations:** 1 Université Clermont Auvergne, INRAE, GDEC, Clermont-Ferrand, France; 2 Centro de Biotecnología y Genómica de Plantas, Universidad Politécnica de Madrid (UPM), Instituto Nacional de Investigación y Technología Agraria y Alimentaria (INIA), Pozuelo de Alarcón, Madrid, 28223 Spain; 3 Université Paris-Saclay, INRAE, UR BIOGER, Thiverval-Grignon, France

**Keywords:** *Zymoseptoria Tritici*, Host resistances, Non-host resistance, *Stb* genes, Wheat, Stomatal penetration, Mesophyll colonization, Functional mechanism, Quantitative cytology

## Abstract

**Background:**

Septoria tritici blotch (STB), caused by the foliar fungus *Zymoseptoria tritici*, is one of the most damaging disease of wheat in Europe. Genetic resistance against this fungus relies on different types of resistance from non-host resistance (NHR) and host species specific resistance (HSSR) to host resistance mediated by quantitative trait loci (QTLs) or major resistance genes (*Stb*). Characterizing the diversity of theses resistances is of great importance for breeding wheat cultivars with efficient and durable resistance. While the functional mechanisms underlying these resistance types are not well understood, increasing piece of evidence suggest that fungus stomatal penetration and early establishment in the apoplast are both crucial for the outcome of some interactions between *Z. tritici* and plants. To validate and extend these previous observations, we conducted quantitative comparative phenotypical and cytological analyses of the infection process corresponding to 22 different interactions between plant species and *Z. tritici* isolates. These interactions included four major bread wheat *Stb* genes, four bread wheat accessions with contrasting quantitative resistance, two species resistant to *Z. tritici* isolates from bread wheat (HSSR) and four plant species resistant to all *Z. tritici* isolates (NHR).

**Results:**

Infiltration of *Z. tritici* spores into plant leaves allowed the partial bypass of all bread wheat resistances and *durum* wheat resistance, but not resistances from other plants species. Quantitative comparative cytological analysis showed that in the non-grass plant *Nicotiana benthamiana*,* Z. tritici* was stopped before stomatal penetration. By contrast, in all resistant grass plants, *Z. tritici* was stopped, at least partly, during stomatal penetration. The intensity of this early plant control process varied depending on resistance types, quantitative resistances being the least effective. These analyses also demonstrated that *Stb*-mediated resistances, HSSR and NHR, but not quantitative resistances, relied on the strong growth inhibition of the few *Z. tritici* penetrating hyphae at their entry point in the sub-stomatal cavity.

**Conclusions:**

In addition to furnishing a robust quantitative cytological assessment system, our study uncovered three stopping patterns of *Z. tritici* by plant resistances. Stomatal resistance was found important for most resistances to *Z. tritici*, independently of its type (*Stb*, HSSR, NHR). These results provided a basis for the functional analysis of wheat resistance to *Z. tritici* and its improvement.

**Supplementary Information:**

The online version contains supplementary material available at 10.1186/s12870-024-05426-5.

## Background

*Zymoseptoria tritici* is a fungal pathogen responsible for the polycyclic foliar disease of wheat, Septoria tritici blotch (STB), one of the most damaging wheat disease in Europe [1, 2]. This genetically highly diverse fungus exhibits a singular biphasic infection with a long asymptomatic phase followed by a necrotrophic phase. The duration of each phase is flexible over time, depending on isolate/wheat genotypes and environmental conditions. The germination of *Z. tritici* spores begins a few hours after their contact with the leaf surface. The resulting hyphae initiate a stochastic epiphytic development that can extend up to 18 days [3–5]. During the exploration of the leaf surface, hyphae attempt to penetrate inside the leaf through the stomata, a process that occurs from 1 to 13 days without any specialized structure such as appressoria [4, 5]. Once inside the leaf, hyphae colonize the apoplast without entering into mesophyll cells. During this colonization, no macroscopic symptoms are observed (asymptomatic phase). After 8 to 18 days, *Z. tritici* differentiate asexual reproduction structures – the pycnidia – by aggregating hyphae in sub-stomatal cavities, which coincides with the appearance of chlorosis and necrosis on the leaf [4, 5]. Finally, mature pycnidia release pycnidiospores on the leaf surface through stomata to initiate another infection cycle.

Genetic resistance, the main environmentally sustainable strategy to control STB, comes in various types. Non-host resistance (NHR) refers to an entire plant species that is resistant to all genetic variants of non-adapted pathogens [6, 7]. Plant species such as *Brachypodium distachyon* [8–10] and *Nicotiana benthamiana* [11] are resistant to all *Z. tritici* isolates tested and are considered as carrying non-host resistance to *Z. tritici*. *Z. tritici* has been reported as a pathogen of few grass species [12], such wheat species as *T. aestivum*, *T. durum*, *T. dicoccum* and *T. compactum*, out of 25 grass species evaluated [13]. However, a given species is in general resistant to *Z. tritici* isolates collected on other species of the genus [14–16]. This resistance to *Z. tritici* could be defined as host species specific resistance (HSSR). While the genetic basis of NHR and HSSR is not well understood, host resistance of wheat specie *T. aestivum* against *Z. tritici* isolates collected on this species has been the most studied types of resistance and is controlled by major *Stb* genes or quantitative trait loci (QTLs). To date, 23 *Stb* genes have been mapped onto the wheat genome [16–18]. Resistance mediated by *Stb* genes, like *Stb6*, are based on a gene-for-gene interaction with a single Avr factor on *Z. tritici* side, and often confer a complete resistance [19–21]. Additionally, more than 300 resistance QTLs have been identified in 89 genomic regions of wheat genome and operate in a quantitative manner, as they only attenuate disease severity [16, 22–24]. Recent reports revealed that these quantitative resistances could also involve gene-for-gene interactions [18, 25].

Understanding the mechanisms controlled by these different types of resistances against *Z. tritici* is of importance to breed efficient and durable STB-resistant wheat cultivars. Only few studies have focused on HSSR and NHR, drawing a very limited picture of those resistance mechanisms. For instance, resistance mediated by *B. distachyon* and *N. benthamiana* have been shown to possibly involve effectors produced by *Z. tritici* [8–11]. By contrast, most functional studies have focused on bread wheat resistance to *Z. tritici*. Comparative -omics have revealed an early upregulation of carbohydrate metabolism, a cell wall reinforcement and the accumulation of defense proteins and possible antifungal metabolites in wheat cultivars resistant to *Z. tritici* [26–30] but no hypersensitive response (HR), nor lignin and polyphenolic depositions [31, 32] have been described in such interactions. Cellular autofluorescence [31, 33, 34], local ROS [29, 34–36] and callose accumulations [31, 34], and more recently, stomatal closure [25, 36, 37] are other responses that have been associated with wheat resistance. However, the role of these various responses in host resistance remains elusive, and their diversity and orchestration are still unknown.

Cytological studies of plant *Z. tritici* interactions have shown that the stomatal penetration and the early establishment of the fungus in the apoplast are critical stages for *Z. tritici* infection outcome in *T. aestivum* [25, 32, 34, 38], *Triticum monococcum* [15] and *B. distachyon* [8]. However, most studies have been performed with a few and/or undefined resistance sources. Furthermore, these studies were performed using different techniques, such as light microscopy with cleared and stained samples [8, 32, 34, 38], confocal laser scanning microscopy with fluorescent dyes or transgenic isolates [4, 19, 25] and scanning/transmission electron microscopy [15, 32, 34] that allow the acquisition of qualitative or semi-quantitative data. Though these methodologies could be useful to describe the impact of the different types of resistances on the infection process, a precise quantitative analysis is still missing.

Recently, a quantitative cytological analysis was performed on near-isogenic wheat lines carrying or not the *Stb16q* gene, one of the three cloned *Stb* genes [19, 39, 40], infected with virulent and avirulent transgenic *Z. tritici* isolates expressing the fluorescent GFP [37]. This study demonstrated that *Stb16q* mainly stops an avirulent *Z. tritici* isolate during stomatal penetration [37]. Likewise, cytological observations of GFP-labelled avirulent *Z. tritici* isolates also highlighted that the *Stb6* gene stops avirulent isolates during their penetration into leaves [41]. In this work, we evaluated whether stomatal penetration is a critical stage during which different types of plant resistance stop *Z. tritici* infection. We focused on resistances to *Z. tritici* mediated by the major genes *Stb5*, *Stb6*, *Stb7* and *Stb9*, and on resistances of contrasting efficiencies of four wheat accessions (Pocho, CDC Landmark, SY Mattis and CDC Stanley). We also analysed the resistance of durum wheat and triticale to *Z. tritici* (HSSR) and resistance of barley, rye, *B. distachyon* and *N. benthamiana* to *Z. tritici* (NHR). By using two inoculation methods, five cytological indexes derived from quantitative cytological analyses with transgenic *Z. tritici* isolates expressing the fluorescent GFP and K-means clustering of these cytological indexes, we identified stopping patterns of *Z. tritici* associated with plant resistance.

## Methods

### Plant and fungal materials

Wheat genotypes and *Z. tritici* isolates used in this study are listed in Table 1. Five wheat Chinese Spring (CS) quasi near isogenic lines (NILs) carrying either no *Stb* gene (NIL^stb^), or *Stb5* (NIL^Stb5^) *Stb6* (NIL^Stb6^), *Stb7* (NIL^Stb7^) and *Stb9* (NIL^Stb9^) were used. NIL^stb^, NIL^Stb6^ and NIL^Stb9^ were described in Battache et al., 2022. NIL^Stb5^ and NIL^Stb7^ were obtained following five backcrosses with the recurrent parent NIL^stb^ starting from F1 between CS and accessions CS-Synthetic and Estanzuela Federal, respectively [42]. At each generation, progenies were genotyped with SSR markers wms044 and wmc405 to follow *Stb5* and with SSR markers wmc313, wmc219 and wmc497 for *Stb7*. The progenies were also phenotyped with *Stb5* IPO94269 avirulent isolate and *Stb7* ISR398 avirulent isolate. A BC_5_F_1_ plant heterozygous for *Stb5* or *Stb7* was self-fertilized. A BC_5_F_2_ carrying the resistance gene *Stb5* and a BC_5_F_2_ carrying the resistant allele of *Stb7* at the homozygous state were selected and named NIL^Stb5^ and NIL^Stb7^, respectively.

Wheat accession Pocho (ERGE 35904), barley cultivar Morex (ERGE 12751) and rye cultivar “Seigle de millevaches” (ERGE 33870) were obtained from the Biological Resource Center on small grains cereals (INRAE, France). Cultivars CDC Landmark (PANG0003), SY Mattis (PANG0015) and CDC Stanley (PANG0004) were obtained from the SeedStor (John Innes Centre, UK). The durum wheat cultivar Svevo was kindly provided by Pasquale De Vita (CREA, Italia) and the triticale cultivar Ramdam was obtanied from AgriObtention (France). *Brachypodium distachyon* ecotype Bd21-3 [43] and *Nicotiana benthamiana* were also included.

*Z. tritici* isolates IPO9415 (virulent on *Stb6*, *Stb7* and *Stb9*) and CFZ008 (virulent on *Stb6* and *Stb9* and avirulent on *Stb5*) were collected in French wheat fields on cultivar Premio in 2009 and cultivar Cellule in 2016, respectively. The *Stb9*-avirulent IPO89011 isolate and the *Stb6*-avirulent IPO323 isolate were collected from Netherlands wheat fields [44, 45]. The *Stb5*-virulent IPO92006 and the *Stb7*-avirulent ISR398 isolates were collected from a Portuguese and an Israeli wheat fields respectively [15, 31]. For cytological analysis, GFP-expressing transformants of all those isolates were obtained using *Agrobacterium tumefasciens*-mediated transformation (ATMT) with pYSKH-4 plasmid, as described in Battache et al., 2022. For cytological analysis on *B. distachyon* and *N. benthamiana*, *Z. tritici* isolate ST99CH_3D7 [46] expressing GFP (3D7-GFP) was used.

**Table 1:** Wheat genotypes and *Z. tritici* isolates used in this study

Wheat genotypes	Types of resistance	*Z. tritici* isolates (virulent/avirulent in the case of stb mediated resistance)
NIL^stb^	Susceptible control	All
NIL^Stb5^	HR mediated by *Stb5*	IPO92006/CFZ008
NIL^Stb6^	HR mediated by *Stb6*	CFZ008/IPO323
NIL^Stb7^	HR mediated by *Stb7*	IPO9415/ISR398
NIL^Stb9^	HR mediated by *Stb9*	CFZ008/IPO89011
Pocho	Quantitative HR	IPO9415/CFZ008
CDC Landmark	Quantitative HR	IPO9415/CFZ008
SY Mattis	Quantitative HR	IPO9415/CFZ008
CDC Stanley	Quantitative HR	IPO9415/CFZ008
Svevo	HSSR	IPO9415
Randam	NHR	IPO9415
Morex	NHR	IPO9415
Seigle de millevaches	NHR	IPO9415
*Brachypodium distachyon* ecotype Bd21-3	NHR	ST99CH_3D7
*Nicotiana benthamiana*	NHR	ST99CH_3D7

### Plant and *Zymoseptoria tritici* growing conditions

All experiments were performed using the attached leaf assay [47] except for cytological analyses conducted on NIL^Stb6^ and NIL^Stb9^ and on *B. distachyon* and *N. benthamiana*. The plants were grown in 60 cm × 40 cm trays filled with Humustar seedling soil (NPK 14-16-18%; SARL Activert, Riom, France) under a 16 h photoperiod at 21/18°C (day/night), either under 90% relative humidity (RH) in a controlled growth chamber equipped with fluorescent tubes (half Master TL-D Super 80 36 W/865 1 SL, half Master TL-D Super 80 36 W/830 UNP; 200 µmol.m^− 2^.s^− 1^; Philips, Amsterdam, Netherlands) (for cytological analysis) or under 80% HR in the MTR30 growth chamber (Conviron^®^) equipped with fluorescent tubes (Master TL-D Super 80 70 W/840; 480 µmol. m^− 2^.s^− 1^; Philips, Amsterdam, Netherlands) (for all other experiments). For cytological analysis conducted on NIL^Stb6^ and NIL^Stb9^, plants were grown in a 10 cm × 10 cm pot filled with the FloradurR B soil (NPK 14-16-18%; Floragard Vertriebs-GmbH, Oldenburg, Germany) in a controlled growth chamber with fluorescent tubes (Osram Lumilux L58W/830; 300 µmol.m^− 2^. s^− 1^; OSRAM GmbH, Munich, Germany), under a 16 h photoperiod at 22/18°C (day/night) and 80% RH. For cytological experiment on *B. distachyon* and *N. benthamiana*, plants were grown in 7 cm × 7 cm pots containing Tray superfine (NPK 155-80-145 mg.L^− 1^; Gramoflor GmbH, Vechta, Germany) in a controlled growth chamber with LED plates (58 µmol.m^− 2^.s^− 1^), under a 16 h photoperiod at 18/15°C (day/night) and 65% HR.

For all experiments except cytological analysis conducted on NIL^Stb6^ and NIL^Stb9^ and on *B. distachyon* and *N. benthamiana*, *Z. tritici* isolates and transformants were grown in liquid YG with 100 mg/L streptomycin and 100 mg/L ampicillin at 20 °C, 180 rpm, for 3 days and spread on YPD plates supplemented with the same antibiotics at 20 °C for 4 days. A suspension of 1.10^6^ spores/mL supplemented with 0.05% (v/v) Tween-20 was prepared to inoculate attached leaves. For cytological analysis conducted on NIL^Stb6^ and NIL^Stb9^, *Z. tritici* IPO323-GFP, IPO89011-GFP and CFZ008-GFP isolates were grown on YPD plates supplemented with 100 mg/L ampicillin at 18 °C, 70–80% RH for 3 days, and spread again on new YPD plates for 4 more days. A suspension of 3.10^6^ spores/ml supplemented with 10% (v/v) gelatine was prepared to inoculate unattached leaves. For cytological analysis conducted on *B. distachyon* and *N. benthamiana*, *Z. tritici* ST99CH_3D7 isolate was grown in liquid YPD with 50 mg/L kanamycin at 18 °C, 120 rpm, for 5 days. A suspension of 1.10^7^ spores/mL supplemented with 0.1% (v/v) Tween-20 was prepared to inoculate unattached leaves.

### Inoculation procedures

For all experiments except cytological analysis conducted on *B. distachyon* and *N. benthamiana*, 6- to 8- centimetre sections of the second leaf of 14-day-old plants were inoculated with a paintbrush six times repeated twice (or 3 times twice for cytological analysis conducted on NIL^Stb6^ and NIL^Stb9^) with spore suspensions or water supplemented with 0.05% (v/v) Tween20 as control solution. Infiltration assays were performed by infiltrating between 0.01 and 0.5 mL of spore suspensions or control solution at three different locations in second leaves using a needle-less syringe, so that a 6- to 8-centimetre section was entirely infiltrated. For cytological analysis conducted on *B. distachyon* and *N. benthamiana*, pots containing 14- and 17-day-old plants, respectively, were spray-inoculated with 5 mL of spore suspension. Following inoculation, the plants were covered with transparent bags for 3 days before returning to normal conditions. Disease severity of inoculated leaves was visually evaluated at 21 dpi by estimating the percentage of the leaf surface covered with symptoms (chlorosis and necrosis) and pycnidia. For phenotyping assays, results were obtained from six individual leaves per condition from two independent experiments.

### Cytological analyses

For all cytological experiments except the ones conducted on NIL^Stb6^ and NIL^Stb9^ and on *B. distachyon* and *N. benthamiana*, a 2-cm section per leaf was harvested at 9 dpi and stained 30 s with 0.1% Calcofluor White M2R (Sigma-Aldrich) in water, briefly rinsed in water, set on slides with double-side adhesive tape and mounted in water. Stained samples were observed using an Axio Observer Z1 (Zeiss) fluorescent microscope with filter set #38 (Zeiss) (ex: 450–490 nm, em: 500–550 nm) and filter set #49 (Zeiss) (ex: 300–400 nm, em: 420–470 nm) to visualize the GFP transgenic fungal line and Calcofluor White M2R, respectively. Five random Z-stack images of 781 × 626 μm for each 2-cm leaf sections were acquired under an EC Plan-Neofluar 10×/0.3 Ph1 M27 objective, 1.6× optovar, using Zeiss Zen 3.1 software (Blue edition). Cytological analysis conducted on NIL^Stb6^ and NIL^Stb9^ were performed at 9 dpi with a Leica DM5500 B fluorescent microscope according to the method described in Battache et al., 2022. For cytological analysis performed on *B. distachyon* and *N. benthamiana*, 4-cm leaf sections or the full leaf respectively were observed at 9 dpi under a LSM 880 (Zeiss) confocal microscope with a GFP filter (ex : 405 nm, em : 511–564 nm) and a UV filter (ex : 488 nm, em : 692–697 nm) to visualize chloroplasts. Three to four random images per leaf section were acquired using the fast Airyscan software.

Images were analysed using Fiji [48] for quantification of the number of stomata reached by epiphytic hyphae, the number of penetration attempts, the number of primary sub-stomatal cavities colonization (or successful penetration events), the number of secondary sub-stomatal cavities colonization and the number of in-formation pycnidia as described in Battache et al., 2022. Five indexes were then calculated: (i) the percentage of reached stomata, i.e. the number of reached stomata relative to the total number of stomata; (ii) the penetration attempt efficiency, i.e. the number of penetration attempts relative to the number of reached stomata; (iii) the penetration success rate, i.e. the number of sub-stomatal cavity primarily colonized relative to the number of penetration attempts; (iv) the secondary colonization efficiency, i.e. the number of sub-stomatal cavities secondarily colonized relative to the number of sub-stomatal cavities primarily colonized and (v) the pycnidia initiation efficiency, i.e. the number of in-formation pycnidia relative to the number of primarily and secondarily colonized sub-stomatal cavities. Results were obtained from six individuals leaves per condition from two independent experiments, except for cytological analysis on *B. distachyon* and *N. benthamiana* where results were obtained from three individuals leaves per condition from one independent experiment. For final comparison, K-means clustering was achieved on data after Z-score normalization (kmeans function from “stats” package in R software (4.1.0 version)).

### Statistical analyses

Statistical analyses were carried out using the R software (4.1.0 version). Data are expressed as mean ± standard error of the mean. Differences in pycnidia and symptoms coverage were analysed using the non-parametric multi-factorial method Aligned Rank Transformation (ART) ANOVA with the ART function of the “ARTool” package and a Tukey’s multiple range test. Statistical analysis of cytological indexes was performed using the one-way non-parametric Van der Waerden test from “agricolae” package, combining genotype (or species in the case of NHR and HSSR) and treatment (*Z. tritici* isolates or control) in a single factor. Due to divergences in experimental conditions/methods between the different cytological experiments, each NIL^stb^ / NIL^Stbx^ pair was statistically analysed independently and data on *B. distachyon* and *N. benthamiana* were excluded from statistical analysis. All *p*-values < 0.05 were considered to be significant.

## Results

### Phenotypic responses of grass accessions carrying different types of resistances against *Z. tritici*

To compare the responses of grasses carrying different types of resistance against *Z. tritici*, we selected wheat quasi near-isogenic lines (NILs) from the Chinese Spring accession, carrying *Stb5* or *Stb7* (NIL^Stb5^ and NIL^Stb7^) along with four wheat accessions (Pocho, CDC Landmark, SY Mattis and CDC Stanley) with phenotypically quantitative resistance to *Z. tritici* (unpublished data). Additionally, we included one accession of four grass species carrying either resistance classified as non-host (rye and barley, NHR) or host species specific to *Z. tritici* isolates collected on bread wheat (durum wheat and triticale, HSSR).

**Fig. 1:**
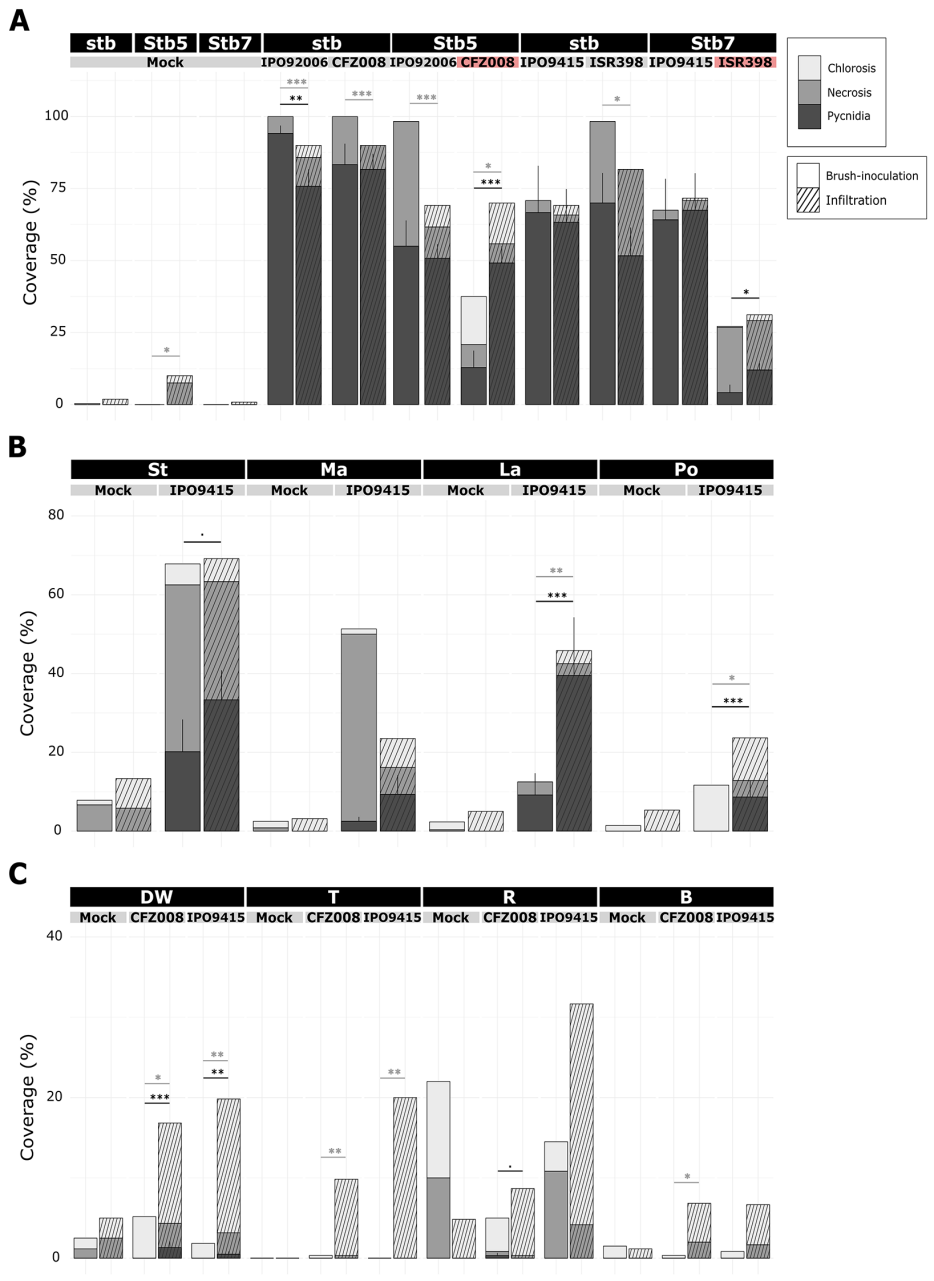
Symptoms after 21 days post-inoculation on plants carrying various types of resistance against *Z. tritici.* Symptoms visually observed at 21 dpi on the second leaves of (**A**) NILs, (**B**) of four wheat accessions and (**C**) of four grass species, inoculated with a control solution (water/Tween20 0.05 (v/v); = Mock) or virulent and avirulent (in red) *Z. tritici* isolates using brush-inoculation or leaf infiltration. Bars represents percentages of leaf surface covered with chlorosis and necrosis (bars stacked), and with pycnidia. Values are means ± SEM [*n* = 6] from two independent experiments. Only SEM for pycnidia are represented. Statistically significant differences are shown in light grey for chlorosis and necrosis, and in dark grey for pycnidia (“.”: 0.1 < *p* < 0.05; “*”: *p* < 0.05; “**”: *p* < 0.01; “***”: *p* < 0.001). stb = NIL^stb^ ; Stbx = NIL^Stbx^ ; St = CDC Stanley ; Ma = SY Mattis ; La = CDC Landmark ; Po = Pocho ; DW = durum wheat ; T = triticale ; R = rye ; B = barley

The NIL lacking any known *Stb* gene (NIL^stb^) and the NIL carrying the *Stb5* gene (NIL^Stb5^) were brush-inoculated with the virulent IPO92006 and the *Stb5*-avirulent *Z. tritici* CFZ008 isolates. Similarly, the NIL lacking any known *Stb* gene (NIL^stb^) and the NIL carrying the *Stb7* gene (NIL^Stb7^) were brush-inoculated with the virulent *Z. tritici* IPO9415 and the *Stb7*-avirulent *Z. tritici* ISR398 isolates. At 21 dpi, NIL^stb^ inoculated with all four isolates exhibited percentages of leaves covered with pycnidia ranging from 67% for the IPO9415 isolate, to 94% for the IPO92006 isolate. The inoculation of *Stb5*-virulent IPO92006 and the *Stb7*-virulent IPO9415 isolates on NIL^Stb5^ and NIL^Stb7^, induced pycnidia covering from 55 to 64% of the leaf surface, respectively. It is noteworthy to notice that NIL^Stb5^ showed 1.7 times less pycnidia than NIL^stb^ when inoculated with the IPO92006 isolate, suggesting the presence of a quantitative resistance in NIL^Stb5^ against this isolate. By contrast, in the incompatible interactions, the *Stb5*avirulent CFZ008 and the *Stb7*-avirulent ISR398 isolates induced pycnidia covering only 13 and 4% of leaf surface of NIL^Stb5^ and NIL^Stb7^, respectively (Fig. 1A, empty bars; Fig. S1A). These findings demonstrated that *Stb5* and *Stb7* mediated strong, but not full, resistance against avirulent CFZ008 and ISR398 isolates, respectively.

In parallel, the four wheat accessions with quantitative resistance were brush-inoculated with the *Z. tritici* IPO9415 isolate. At 21 dpi, only 12% of the inoculated leaf surface from landrace Pocho displayed chloroses without pycnidia. Leaves from cultivars CDC Landmark and SY Mattis displayed 9% and 3% of their inoculated surface with pycnidia with 12% and 50% of leaf necrosis, respectively. Cultivar CDC Stanley was the most susceptible, as 20% and 62% of the inoculated leaf surfaces carried pycnidia or necrosis, respectively (Fig. 1B, empty bars ; Fig. S1B). These results showed that the four wheat accessions displayed different resistance efficiency against the IPO9415 isolate, ranging from the fully resistant accession Pocho, to the moderately susceptible cultivar CDC Stanley, with cultivars CDC Landmark and SY Mattis showing intermediate resistances.

The four grass species (rye, barley, durum wheat and triticale) were inoculated with *Z. tritici* IPO9415 and CFZ008 isolates, collected from bread wheat. Except for rye, which leaves exhibited rare pycnidia (< 0.33% of leaf surface) when inoculated with the CFZ008 isolate, none of the inoculated leaves from other grasses displayed pycnidia, nor more chlorosis and necrosis than control plants (Fig. 1C, empty bars ; Fig. S1C). These results indicated that these grasses displayed full resistance against *Z. tritici*.

### Infiltration of *Z. tritici* into leaves partially bypass *triticum* resistances but not NHR

Similar to brush-inoculations, infiltration of *Z. tritici* spores were performed on leaves of the same genetic materials. Except for the compatible interaction between the IPO92006 isolate and NIL^stb^, for which the infiltrated leaves showed significantly 18% less pycnidia than the brush-inoculated leaves, all other compatible interactions displayed inoculated leaves with similar amount of pycnidia when comparing infiltration and brush-inoculation at 21 dpi. By contrast, in the incompatible interactions, inoculated leaves of NIL^Stb5^ and NIL^Stb7^ showed 3.8 and 3 times more pycnidia after infiltration than after brush-inoculation with the avirulent CFZ008 and ISR398 isolates, respectively. However, in these two interactions, pycnidia were roughly restricted to the infiltration spots and did not extend as on NIL^stb^ infiltrated with the same isolates (Fig. 1A, stripped bars; Fig. S1A). The inoculated leaves from the four wheat accessions, CDC Stanley, SY Mattis, CDC Landmark and Pocho showed respectively 1.6, 3.72, 4.3 and 8.7 more pycnidia when infiltrated with the IPO9415 isolate compared to brush-inoculation (Fig. 1B, stripped bars, significant for CDC Landmark and Pocho ; Fig. S1B). For the other grasses, while infiltration induced more chlorosis and necrosis overall than brush-inoculation, a few pycnidia developed only on leaves of durum wheat infiltrated with the IPO9415 isolate (Fig. 1C, stripped bars; Fig. S1C). Together, these results showed that the use of an inoculation method circumventing the penetration process enabled *Z. tritici* to bypass, at least partially, all bread wheat resistances tested and durum wheat resistance. These results suggested that the control of *Z. tritici* penetration was critical for these types of resistances. These results also showed that the infiltration method did not allow *Z. tritici* to infect rye, barley and triticale, suggesting that these resistances did not rely, or not only/mainly, on the control of *Z. tritici* penetration into leaves.

### The epiphytic stage of *Z. tritici* is affected by some NHR and HSSR

To investigate more precisely where and when *Z. tritici* was stopped during its infection of plants with different types of resistance, we used GFP-labelled *Z. tritici* isolates from bread wheat, virulent or avirulent on *Stb5*- and *Stb7-*NILs pairs, virulent on the four wheat accessions and avirulent on durum wheat, triticale and four other resistant plant species. The infection processes of these transgenic isolates were monitored at 9 dpi using epifluorescence microscopy. NIL^Stb6^ and NIL^Stb9^, mediating full resistance against *Z. tritici* IPO323 and IPO89011 isolates respectively [37], were also included in this analysis. The fungal chitin dye calcofluor was used to stain the surface of inoculated leaves. This staining enabled epiphytic hyphae (GFP-fluorescent and calcofluor-stained) to be distinguished from infectious hyphae (only GFP-fluorescent). Additionally, more distantly related species including the grass *Brachypodium distachyon* and the dicotyledon *Nicotiana benthamiana* were also inoculated with a GFP-labelled *Z. tritici* isolate and observed by confocal microscopy. Five cytological indexes were calculated to quantitatively evaluate the efficiency of each *Z. tritici* infectious stages observed at 9 dpi (the percentage of reached stomata, the penetration attempt efficiency, the penetration success rate, the secondary colonisation efficiency, the pycnidia initiation efficiency).

**Fig. 2:**
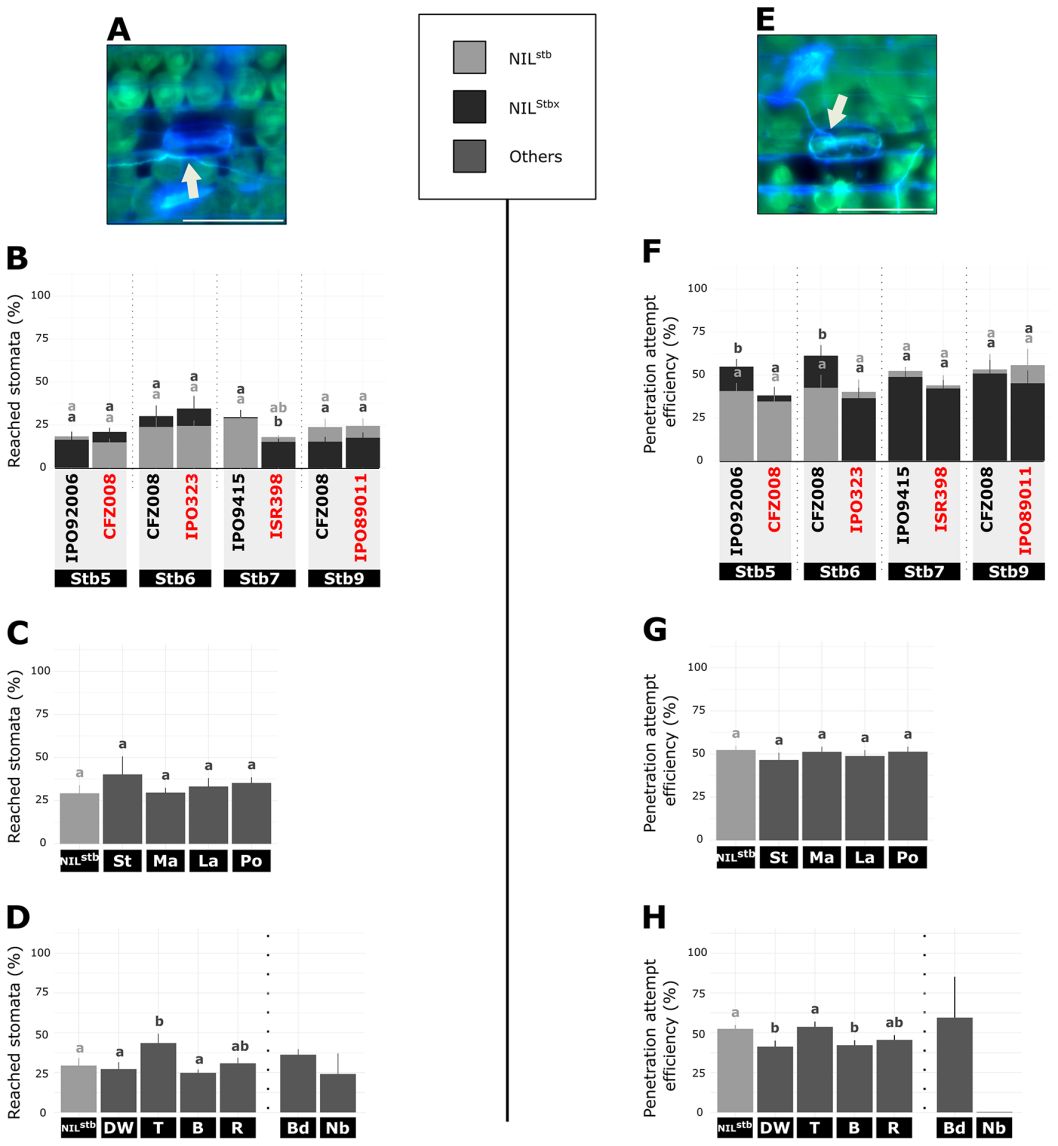
Quantitative cytological assessment of the epiphytic stage of *Z. tritici* on plants carrying different resistances. (**A**) and (**E**), Representative epifluorescence microscopy images of (**A**) a reached stomata and (**E**) a penetration attempt, on wheat leaf inoculated with GFP-labelled isolate stained with the fungal surface-dye calcofluor. White arrow indicates hyphae on leaf surface (green and blue). Bar = 100 μm. (**B**) - (**D**), Percentage of reached stomata at 9 dpi (= number of reached stomata relative to the total number of stomata) and (**F**) - (**H**), Penetration attempt efficiency at 9 dpi (= number of stomata with a penetration attempt relative to the number of reached stomata). (**B**) and (**F**), the NIL^stb^ and four NIL^Stbx^ carrying *Stb5*, *Stb6*, *Stb7* or *Stb9* were inoculated with virulent or avirulent (red) GFP-labelled isolates. (**C**) and (**G**), different wheat accessions and (**D**) and (**H**)¸ five different grass species and a dicot plant were inoculated with the IPO9415 GFP-labelled isolate. Values are means ± SEM [*n* = 6]. Dark grey and light grey letters indicate significantly different values for NIL^Stbx^ and NIL^Stb^, respectively (Van der Waerden test, *p* < 0.05). Vertical dotted bars represent independent statistical analyses as the experiment were performed with slightly different protocols. stb = NIL^stb^ ; Stbx = NIL^Stbx^ ; St = CDC Stanley ; Ma = SY Mattis ; La = CDC Landmark ; Po = Pocho ; DW = durum wheat ; T = triticale ; R = rye ; B = barley

To evaluate the epiphytic stage, the percentage of reached stomata, (Fig. 2A) and the penetration attempt efficiency, (Fig. 2E) were calculated. For each of the four NILs pair, an average of 22% ± 5% of reached stomata were observed, independently of the type of interaction (Fig. 2B). No difference in penetration attempt efficiency was observed between compatible and incompatible interactions on NIL^Stb5^, NIL^Stb6^, NIL^Stb7^ and NIL^Stb9^ (average 47% ± 8%, Fig. 2F). A similar percentage of reached stomata (average 34% ± 3%) and penetration attempt efficiency (average 49% ± 2%) were observed when comparing the four wheat accessions inoculated with the IPO9415 isolate with each other or with NIL^stb^ inoculated with same isolate (Fig. 2C and G). These results indicated that *Stb*-mediated and quantitative resistances do not negatively impact *Z. tritici* epiphytic development.

Regarding other species than *T. aestivum*, all accessions inoculated with the avirulent IPO9415 isolate, as well as *B. distachyon* and *N. benthamiana* inoculated with the avirulent 3D7 isolate showed similar or higher (triticale) percentages of reached stomata than the bread wheat NIL^stb^ inoculated with the virulent IPO9415 isolate (Fig. 2D). Triticale, rye and *B. distachyon* displayed a similar penetration attempt efficiency compared to the bread wheat NIL^stb^ inoculated with the virulent IPO9415 isolate. By contrast, durum wheat and barley accessions showed a significantly lower penetration attempt efficiency (-10%) than observed with the NIL^stb^. *N. benthamiana* showed no penetration attempt at all (Fig. 2H). These results indicated that *Z. tritici* development on the leaf surface was negatively impacted by some of the species displaying NHR or HSSR.

### All types of resistance to *Z. tritici* negatively impact the success of stomatal penetration

**Fig. 3:**
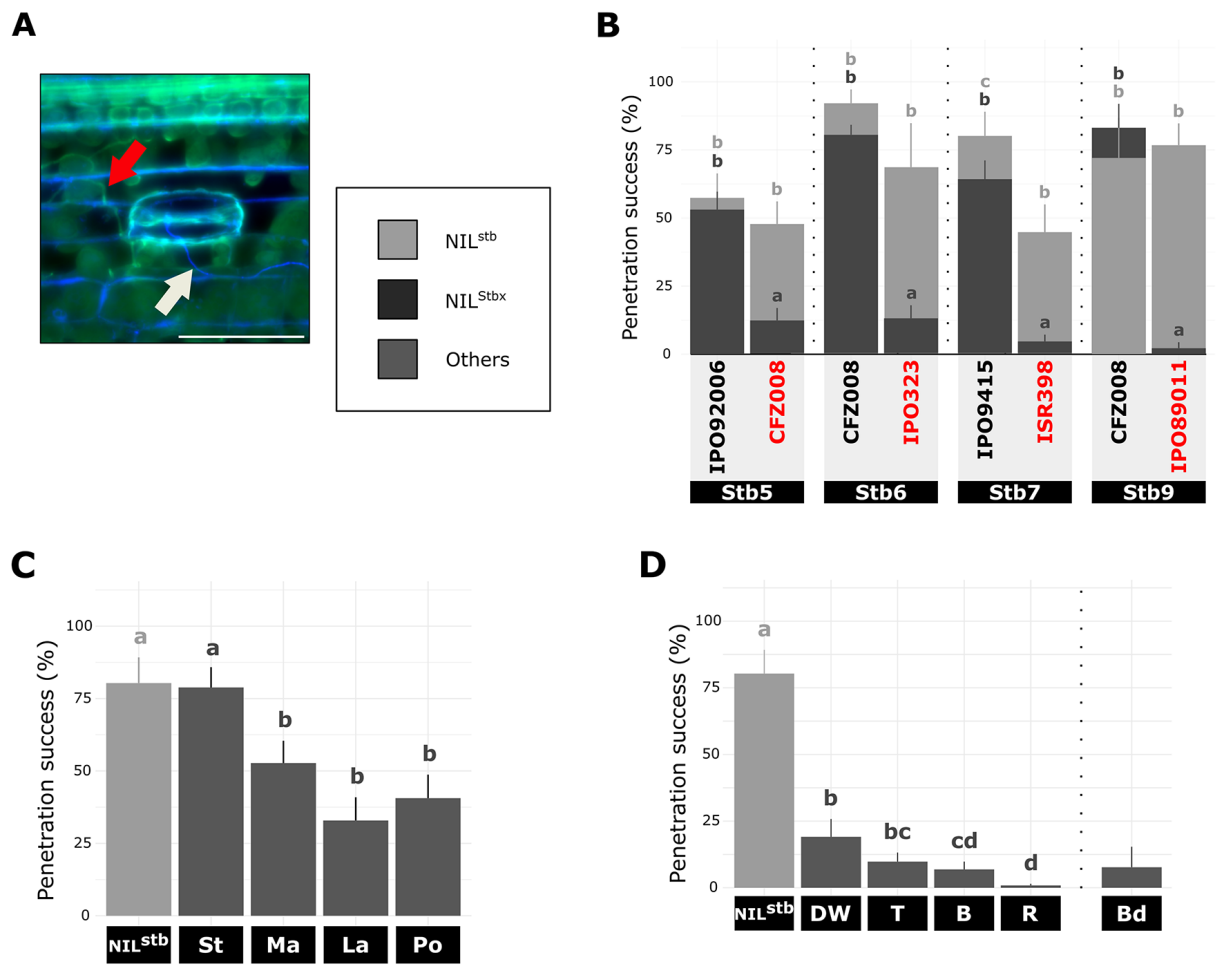
Quantitative cytological assessment of the penetration stage of *Z. tritici* on plants carrying different resistances. (**A**), Representative epifluorescence microscopy image of primarily colonized sub-stomatal cavity (successful penetration event) on wheat leaf inoculated with GFP-labelled isolate stained with the fungal surface-dye calcofluor. White and red arrows indicate hyphae on leaf surface (green and blue) and internal hyphae (only green), respectively. Bar = 100 μm. (**B**) – (**D**), Penetration success rate at 9 dpi (= number of primarily colonized sub-stomatal cavities relative to the number of stomata with a penetration attempt) of (**B**), NIL^stb^ and four NIL^Stbx^ carrying *Stb5*, *Stb6*, *Stb7* or *Stb9* inoculated with virulent or avirulent (red) GFP-labelled isolates, (**C**) different wheat accessions and (**D**) five different grass species inoculated with the IPO9415 GFP-labelled isolate. Values are means ± SEM [*n* = 6]. Dark grey and light grey letters indicate significantly different values for NIL^Stbx^ and NIL^Stb^, respectively (Van der Waerden test, *p* < 0.05). Vertical dotted bars represent independent statistical analyses as the experiment were performed with slightly different protocols. stb = NIL^stb^ ; Stbx = NIL^Stbx^ ; St = CDC Stanley ; Ma = SY Mattis ; La = CDC Landmark ; Po = Pocho ; DW = durum wheat ; T = triticale ; R = rye ; B = barley

The penetration stage was evaluated by calculating the penetration success rate (Fig. 3A). At 9 dpi, the penetration success rate was similar when comparing the three compatible interactions within each NILs pair (53% ± 5% for *Stb5*-NILs pair, 80% ± 12% for *Stb6*-NILs pair and 77% ± 6% for *Stb9*-NILs pair ; Fig. 3B), except for the *Stb7*-NILs pair in which IPO9415/NIL^stb^ compatible interaction presented a higher penetration success (80%) than IPO9415/NIL^Stb7^ and ISR398/NIL^stb^ compatible interactions (64% and 45% ; Fig. 3B). By contrast, in CFZ008/NIL^Stb5^, IPO323/NIL^Stb6^, ISR398/NIL^Stb7^ and IPO89011/NIL^Stb9^ incompatible interactions only 12%, 13%, 5% and 2% penetration success rates were observed, respectively. This represented 3.8, 5.2, 9.5 and 35 less penetration success than the same isolates on NIL^stb^ (Fig. 3B).

Compared to the NIL^stb^, the IPO9415 isolate showed a similar penetration success rate of 79% when inoculated on cultivar CDC Stanley. By contrast, the penetration success rate was 33%, 53% and 41% on cultivars CDC Landmark, SY Mattis and Pocho, respectively, which represented 2.4, 1.5 and 1.97 times less penetration success than observed on NIL^stb^ inoculated with same isolate (80% ; Fig. 3C).

Regarding the different resistant grass species, a maximum penetration success rate of 19% was observed for the IPO9415 isolate on durum wheat, which represents 4.18 times less than observed on NIL^stb^ inoculated with same isolate. This rate decreased to 7%, 10% and 1% on barley, triticale and rye inoculated with the avirulent IPO9415 isolate, respectively, that was 11.6, 8.2 and 89 times less than on NIL^stb^ inoculated with same isolate. Finally, *B. distachyon* inoculated with the 3D7 isolate showed a penetration success rate of 8% (Fig. 3D).

These findings demonstrated that all resistant bread wheat cultivars and grass plants stopped *Z. tritici* during stomatal penetration, with the exception of the bread wheat cultivar CDC Stanley that did not. These results also suggested that the efficiency in stopping *Z. tritici* during stomatal penetration varied according to the resistance types, with quantitative resistances being overall the least effective. By contrast *Stb5-*,* Stb6-*, and *Stb9*-mediated resistance, durum wheat and triticale HSSR, barley and rye NHR were increasingly effective in stopping *Z. tritici* during stomatal penetration.

### Mesophyll colonization is stopped early during *stb*-mediated resistance, HSSR and NHR

**Fig. 4:**
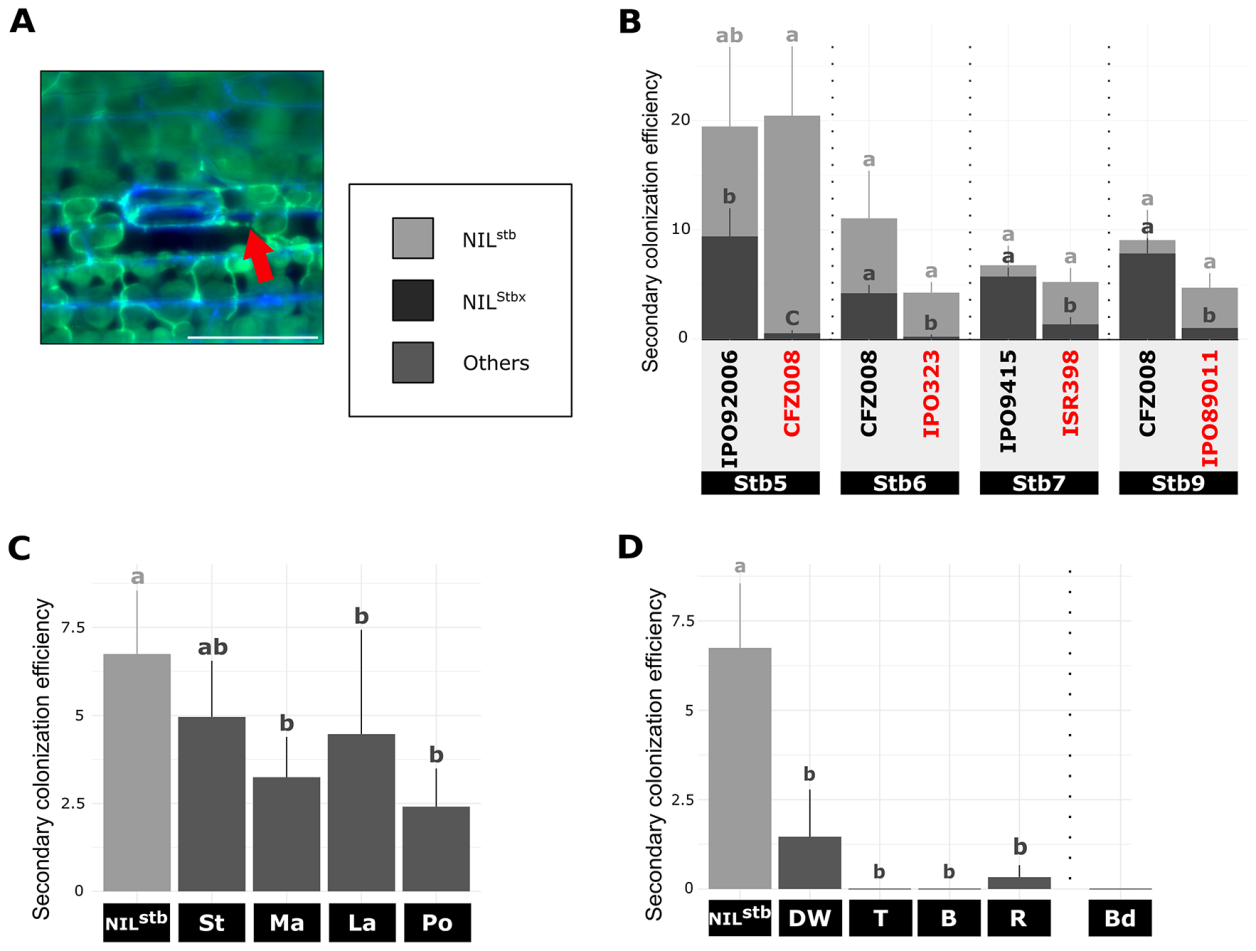
Quantitative cytological assessment of the colonisation stage of *Z. tritici* on plants carrying different resistances. (**A**), Representative epifluorescence microscopy image of a sub-stomatal cavity secondarily colonized on wheat leaf inoculated with GFP-labelled isolate stained with the fungal surface-dye calcofluor. Red arrow indicates internal hyphae (only green). Bar = 100 μm. (**B**) – (**D**), Secondary colonization efficiency at 9 dpi (= number of secondarily colonized sub-stomatal cavities relative to the number of primarily colonized sub-stomatal cavities) of (**B**), NIL^stb^ and four NIL^Stbx^ carrying *Stb5*, *Stb6*, *Stb7* or *Stb9* inoculated with virulent or avirulent (red) GFP-labelled isolates, (**C**) different wheat accessions and (**D**) five different grass species inoculated with the IPO9415 GFP-labelled isolate. Values are means ± SEM [*n* = 6]. Dark grey and light grey letters indicate significantly different values for NIL^Stbx^ and NIL^Stb^, respectively (Van der Waerden test, *p* < 0.05). Vertical dotted bars represent independent statistical analyses as the experiment were performed with slightly different protocols. stb = NIL^stb^ ; Stbx = NIL^Stbx^ ; St = CDC Stanley ; Ma = SY Mattis ; La = CDC Landmark ; Po = Pocho ; DW = durum wheat ; T = triticale ; R = rye ; B = barley

The mesophyll colonisation stage was evaluated by calculating the secondary colonization efficiency (Fig. 4A). For *Stb7*- and *Stb9*-NILs pairs, no difference in secondary colonization efficiency was observed between the three compatible interactions, with hyphae colonizing from 4.7 to 9 sub-stomatal cavities after penetration (Fig. 4B). For *Stb6*- and *Stb5*-NILs pairs, though not significant, the virulent IPO92006 and CFZ008 isolates colonized on average two times fewer sub-stomatal cavities on NIL^Stb5^ (9.4) and NIL^Stb6^ (4.2) respectively than on NIL^stb^ (19.4 and 11, respectively, Fig. 4B), suggesting an impact of *Stb5* and *Stb6* on the colonization of IPO92006 and CFZ008 isolates. By contrast, CFZ008/NIL^Stb5^, IPO323/NIL^Stb6^, ISR398/NIL^Stb7^, IPO89011/NIL^Stb9^ incompatible interactions showed only on average 0.5, 0.2, 1.3 and 1 secondary colonization of sub-stomatal cavities, respectively (Fig. 4B). These results indicated that in all incompatible interactions, the few penetrating hyphae of the avirulent isolates were restricted to their entry point, the first colonized sub-stomatal cavity.

By 9 dpi, penetrating hyphae of the IPO9415 isolate have colonized an average of 6.7 sub-stomatal cavities on NIL^stb^ (Fig. 4C). Similarly, 5 sub-stomatal cavities were colonized on the cultivar CDC Stanley (Fig. 4C). The secondary colonization efficiency was slightly lower on the three other bread wheat accessions, with 3.2, 4.5 and 2.4 sub-stomatal cavities secondarily colonized on SY Mattis, CDC Landmark and Pocho, respectively (Fig. 4C). These results suggested that mesophyll colonization was only slightly impacted by these quantitative resistances.

For the other grass species, secondary colonization was only observed on durum wheat (1.3 sub-stomatal cavities) and on rye (0.33 sub-stomatal cavities) (Fig. 4D), which demonstrated that, similar to *Stb-*mediated resistances, NHR and HSSR prevented avirulent isolates to colonize the mesophyll.

### All types of resistances stopped the infectious hyphae early during fungal reproduction stage

**Fig. 5:**
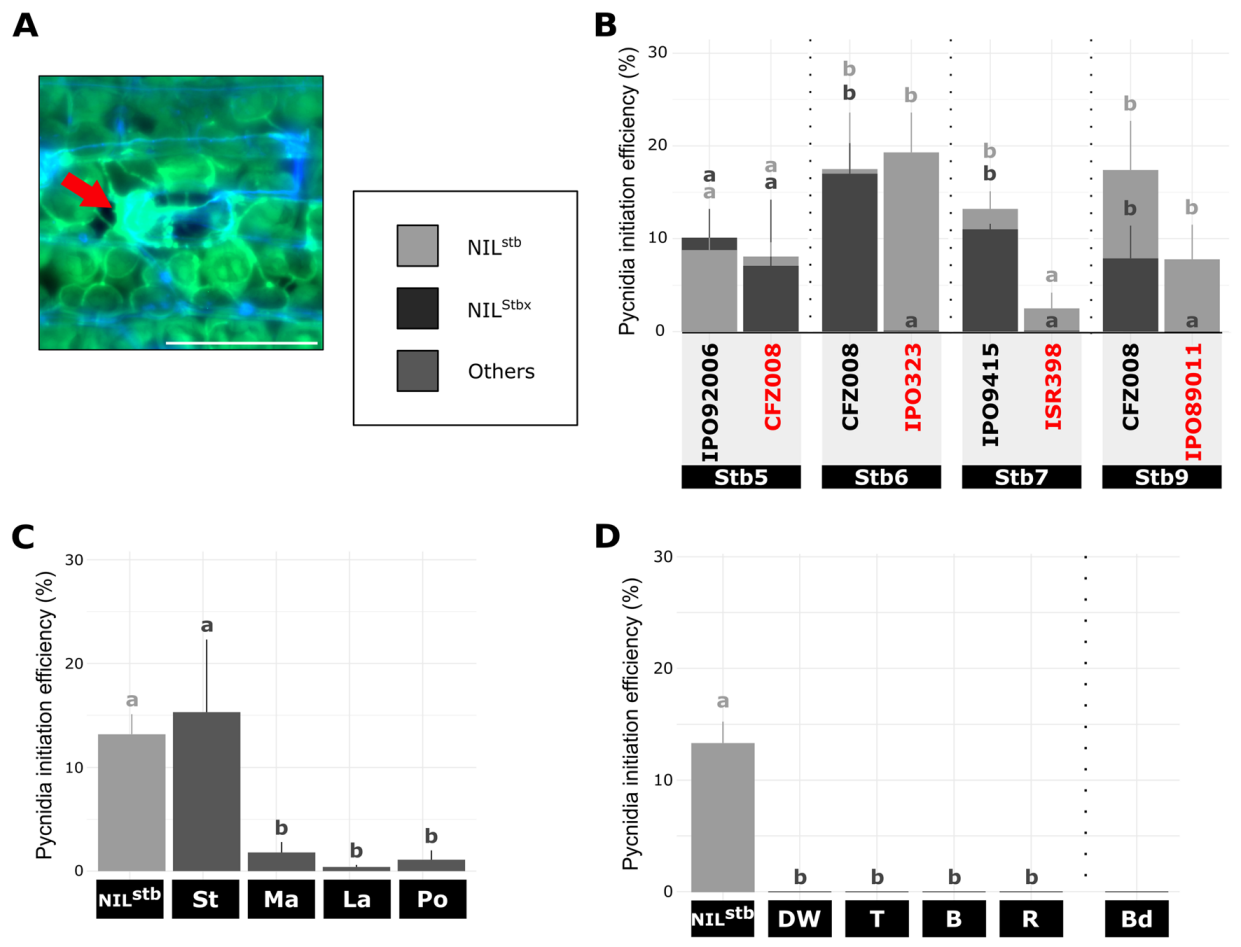
Quantitative cytological assessment of the reproduction stage of *Z. tritici* on plants carrying different resistances. (**A**), Representative epifluorescence microscopy image of in-formation pycnidia on wheat leaf inoculated with GFP-labelled isolate stained with the fungal surface-dye calcofluor at 9 dpi. Red arrow indicates internal hyphae (only green). Bar = 100 μm. (**B**) – (**D**), Pycnidia initiation efficiency at 9 dpi (= number of sub-stomatal cavities bearing in-formation pycnidia relative to the number of primarily and secondarily colonized sub-stomatal cavities) of (**B**), NIL^stb^ and four NIL^Stbx^ carrying *Stb5*, *Stb6*, *Stb7* or *Stb9* inoculated with virulent or avirulent (red) GFP-labelled isolates, (**C**) different wheat accessions and (**D**) five different grass species inoculated with the IPO9415 GFP-labelled isolate. Values are means ± SEM [*n* = 6]. Dark grey and light grey letters indicate significantly different values for NIL^Stbx^ and NIL^Stb^, respectively (Van der Waerden test, *p* < 0.05). Vertical dotted bars represent independent statistical analyses as the experiment were performed with slightly different protocols. stb = NIL^stb^ ; Stbx = NIL^Stbx^ ; St = CDC Stanley ; Ma = SY Mattis ; La = CDC Landmark ; Po = Pocho ; DW = durum wheat ; T = triticale ; R = rye ; B = barley

The reproduction stage was evaluated by calculating the pycnidia initiation efficiency at 9 dpi (Fig. 5A). For the *Stb5*-NILs pair, pycnidia initiation was observed at 9 dpi in average in 8.5% of invaded sub-stomatal cavities independently of the type of interaction, suggesting no impact of *Stb5* on pycnidia initiation. Likewise, no significant difference in pycnidia initiation efficiency was observed at 9 dpi between the ISR398/NIL^Stb7^ incompatible interaction and the ISR398/NIL^stb^ compatible interaction. By contrast, while in average 16% ± 1.8% and 11% ± 4.2% of invaded sub-stomatal cavities showed pycnidia initiation during compatible interactions of *Stb6*- and *Stb9*-NILs pairs respectively, no pycnidia formation was initiated during incompatible interactions on these two NILs at 9 dpi (Fig. 5B). The cultivar CDC Stanley inoculated with the IPO9415 isolate showed the same pycnidia initiation efficiency of 15% as that observed with NIL^stb^ inoculated with the same isolate. By contrast, the three other wheat accessions with quantitative resistance displayed pycnidia initiation efficiencies of less than 1.8% (Fig. 5C). Finally, no pycnidia initiation was observed on the few invaded sub-stomatal cavities of other resistant grass plants (Fig. 5D). These results suggested that all resistances, with the exception of the *Stb5*- and *Stb7*-mediated resistances against CFZ008 and ISR398 isolates, strongly inhibited the formation of pycnidia by the few remaining *Z. tritici* hyphae colonizing sub-stomatal cavities.

### Diversity in *Z. tritici* stopping patterns across different types of resistance

Resistances to *Z. tritici* were classified according to their types (Stb-mediated, quantitative, HSSR, NHR), their phenotypes observed after brush-inoculation of bread wheat *Z. tritici* isolates (IPO09415 and CFZ008), and on their abilities to stop the pathogen at different stages of the infection cycle as observed at 9 dpi. These different parameters were used to produce a three-dimensional plot that integrate all this information to identify stopping patterns associated with the resistance tested and the phenotypic responses observed. According to phenotyping data, the 20 interactions between grasses and *Z. tritici* were classified into four resistance efficiencies: (i) full resistance with no pycnidia, (ii) moderate resistance with few pycnidia (< 15% of leaf surface), (iii) moderate susceptibility with intermediate pycnidia number (between 15 and 75% of leaf surface) and (iv) full susceptibility with many pycnidia (> 75% of leaf surface).

The three most discriminant cytological indexes, the penetration success rate, the secondary colonisation efficiency and the pycnidia initiation efficiency were selected to quantify the effect of plant resistance on *Z. tritici* infection. A clustering analysis of the different patterns revealed four clusters.

**Fig. 6:**
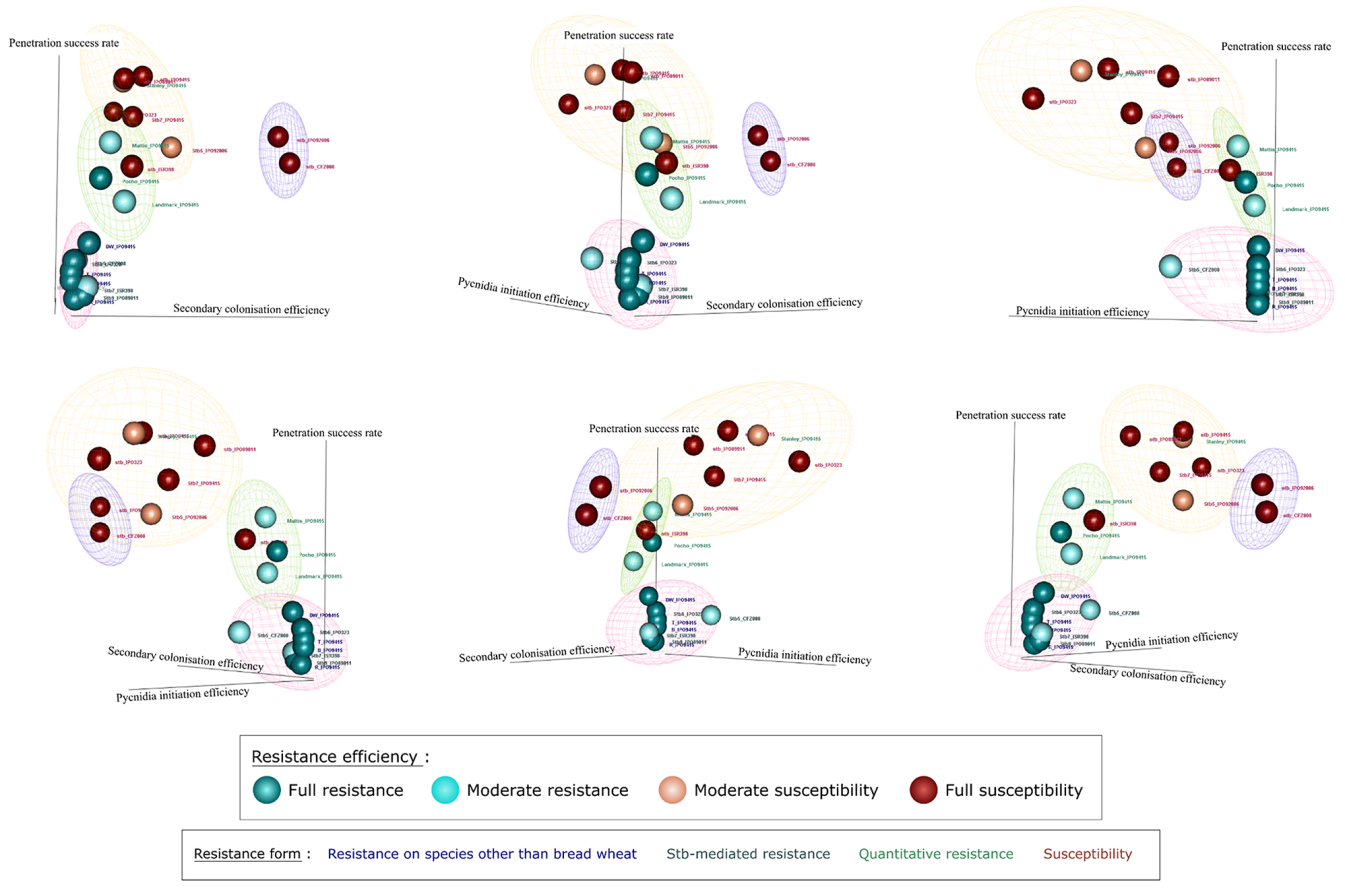
Comparison of the various interactions between grasses and *Z. tritici*. Compilation of the penetration success rate, the secondary colonisation efficiency and the pycnidia initiation efficiency according to the 20 tested interactions between grasses and *Z. tritici*. The six figures represent different 3D view of the clusters. Sphere colours represent the resistance efficiency defined from the phenotypical assays. Text colours indicate the resistance type. “DW” = durum wheat, “T” = triticale, “B” = barley and “R” = rye. “stb_X” represent interactions with the NIL^stb^ and the X isolate, “Stbi_X” represent interactions with the NIL^Stbi^ and the X isolate. Pink (cluster 1), green (cluster 2), purple (cluster 3) and purple (cluster 4) ellipsoids represent K-means clustering after Z-score normalization

The first cluster was characterized by a low penetration success rate (< 20%), a low secondary colonisation efficiency (< 1.3) and a low pycnidia initiation efficiency (< 7%) at 9 dpi (Fig. 6, pink). This cluster included all full and two moderate resistances in terms of phenotyping responses, and all NHR, *Stb-*mediated resistances and HSSR in terms of resistance types. This pattern suggests that these different resistance types involved a similar plant response leading to a main stop of *Z. tritici* infection during stomatal penetration. Although included in this cluster, moderate *Stb5*-mediated resistance against the CFZ008 isolate slightly differed by its higher pycnidia initiation efficiency, suggesting that this weakness is responsible for the quantitative aspect of its resistance phenotype.

The second cluster was characterized by an intermediate penetration success rate (between 33% and 53%), an intermediate secondary colonisation efficiency (between 2.4 and 4.5) and a low pycnidia initiation efficiency (< 2.5%) (Fig. 6, green). This cluster included one full and two moderate resistances carried by wheat accessions Pocho, CDC Landmark and SY Mattis, respectively. This pattern suggested that quantitative resistance cannot rely only on stopping the fungus at the penetration stage. The interaction between NIL^stb^ and the virulent ISR398 isolate also belonged to this second cluster. Its presence with interactions corresponding to quantitative resistances while inducing full susceptibility could reflect a delay in the infection process of this particular isolate.

The third and fourth clusters were characterized by a high penetration success rate (> 45%), a high secondary colonisation efficiency (> 4.7) and a high pycnidia initiation efficiency (> 7.8%) at 9 dpi (Fig. 6, purple and yellow). These clusters gathered all the interactions leading to moderate and full susceptibility, with the exception of the NIL^stb^/ISR398 interaction that belonged to cluster two. The IPO92006/NIL^stb^ and the CFZ008/NIL^stb^ interactions from cluster three (Fig. 6, purple) differed from the interactions of the fourth cluster (Fig. 6, yellow) by a higher secondary colonisation efficiency (> 19), possibly reflecting a greater ability of these two particular isolates to colonize mesophyll compared to other isolates tested. The fourth cluster contained four interactions leading to full susceptibility and two leading to moderate susceptibility. The IPO9415/CDC Stanley interaction, leading to moderate susceptibility clustered with the IPO9415/NIL^stb^ interaction, leading to full susceptibility, suggesting that the differences in their resistance efficiencies could not be explained by these cytological indexes at 9 dpi. By contrast, the IPO92006/NIL^stb^ interaction, leading to full susceptibility, and the IPO92006/NIL^Stb5^ interaction, leading to moderate susceptibility, from the third and fourth clusters respectively, differed only for their secondary colonisation efficiency. This observation suggested that the difference between these two interactions relied on the inhibition of secondary colonisation of the IPO92006 isolate by a *Stb7*-mediated mechanism.

This analysis identified three stopping patterns associated with plant resistance but also differences in the ability of virulent *Z. tritici* isolates to colonize wheat accessions.

## Discussion

Different types of resistance to *Z. tritici* have been described over the last 40 years, from the poorly characterized NHR and HSSR, to the more extensively studied resistances mediated by QTL and major *Stb* genes [8–11, 15–18, 22, 23]. These resistances have been studied independently using various qualitative or semi-quantitative cytological analyses [8, 15, 25, 32, 34, 38, 41], but have never been compared. In this work, through phenotypical and quantitative cytological analysis, we analysed 22 interactions between *Z. tritici* and different plant species. These interactions include (i) NHR mediated by the grass barley, rye and *B. distachyon* and by the dicot *N. benthamiana*, (ii) HSSR mediated by durum wheat and triticale, (iii) quantitative resistances of contrasting efficiency carried by Pocho, CDC Landmark, SY Mattis and CDC Stanley bread wheat accessions and (iv) resistances mediated by major bread wheat genes *Stb5*, *Stb6*, *Stb7* and *Stb9*.

### *N. benthamiana* NHR is associated with a lack of *Z. tritici* penetration attempts

*Z. tritici* was able to develop epiphytically on the surface of all tested plant species in a similar way (25% of stomata reached by epiphytic hyphae). This plant species-independent epiphytic growth is similar to results from previous studies showing that *Z. tritici* was able to grow epiphytically on *B. distachyon* [8–10] and *N. benthamiana* [11] leaves. However, our results showed that *Z. tritici* infection of *N. benthamiana* stopped before hyphae attempt to penetrate through stomata, as not a single penetration attempt was observed. Similarly, Kettles et al. noticed an inhibition of epiphytic growth at 8 dpi after inoculation of *N. benthamiana* with the GFP-expressing B3 *Z. tritici* isolate, with a reduced hyphal growth compared to wheat and only a few reached stomata and/or penetration attempt events, as the study did not differentiate hyphae which reached stomata or attempted to penetrate [11]. Therefore, NHR mediated by *N. benthamiana* seems to rely mainly on the ability of *Z. tritici* to recognize and/or penetrate through stomata.

Previous cytological studies have suggested that *Z. tritici* development on the wheat leaf surface does not follow a directional tropism toward the stomata [3, 4, 32, 34, 38]. Consequently, stomatal recognition preceding the penetration attempt is likely localized at the stomata level. Many filamentous organisms are able to orient themselves and to react depending on the plant surface environment using chemotropism [49–51]. The chemical composition and arrangement of guard cells composing the stomata, and more generally of cell walls, varies considerably between dicots and grass [52–54]. The absence of some specific chemical signals, yet to be determined, at the stomata level could explain the lack of interaction between *N. benthamiana* stomata and *Z. tritici* hyphae. At the same time, one of the most common tropisms of filamentous organisms is the sensing of the leaf relief by thigmotropism [55–57]. For instance, rust fungi of *Uromyces spp.* recognize the stomata and differentiate appressoria through the sensing of a defined surface topology, corresponding to the guard-cells height [55]. In addition to chemical composition, dicot and grass leaf surfaces differ in many structural aspects [58, 59]. One of the main differences between these two groups of plants is found in the stomatal structure, with the presence of two additional cells, called subsidiary cells, surrounding the guard cells in all grass species [54, 59, 60]. It has recently been shown that wheat subsidiary cells were the first wheat cells of the epidermis to react to *Z. tritici* by forming cell wall papillae spatially correlated with the point of fungus contact [61]. This response was not associated with resistance, as cell wall papillae appeared in both compatible and incompatible interactions, and the authors suggested that it is a general response of wheat to *Z. tritici*. This means that *Z. tritici* may recognize subsidiary cells, for instance using thigmotropism, and subsequently initiate the penetration process through molecular or physiological changes, that would in turn induce a wheat subsidiary cells response. The absence of subsidiary cells on dicot is a possible explanation of the lack of interaction between *Z. tritici* epiphytic hyphae and *N. benthamiana* stomata. The elucidation of the exact role of subsidiary cells during *Z. tritici* penetration stage is needed to test this hypothesis. This could be achieved by studying the fungus infection process on other dicot species and on mutant grass lacking subsidiary cells such as the subsidiary cells defective mutant *sid* of *B. distachyon* [62]. Modifying the recognition of these stomata structures could provide interesting approaches to control STB disease.

### Stomatal penetration control: a common key stage for resistances against *Z. tritici*

The cytological analysis of the different stages of *Z. tritici* infection was performed using five quantitative cytological indexes. This analysis revealed three patterns of infection associated with resistance to *Z. tritici*. Pattern 1 was characterized by a main stop of *Z. tritici* at the penetration stage, as evidenced by a low penetration success rate (< 20% vs. > 45% for interactions leading to susceptibility), associated with a strong inhibition of the secondary colonization of sub-stomatal cavities by the few penetrating hyphae (< 1.3 vs. > 4.7 for interactions leading to susceptibility). Pattern 2 relied on a quantitative inhibition of the stomatal penetration (33% < penetration success rate < 53%, vs. > 45% for interactions leading to susceptibility) followed by a relatively efficient colonisation of sub-stomatal cavities (between 2.4 and 4.5, vs. > 4.7 for interactions leading to susceptibility) by penetrating hyphae before being arrested early during pycnidia formation (< 2.5% vs. > 7.8% for interactions leading to susceptibility). Pattern 3 relied on a partial inhibition of the fungus during mesophyll colonisation corresponding to a reduction in the number of secondarily colonized sub-stomatal cavities (9.4 for interaction leading to moderate susceptibility vs. 19.4 for interaction leading to full susceptibility).

All full to moderate resistances analysed here, independently of the resistance types, involved a strong inhibition of fungal penetration, corresponding to interactions from patterns 1 or 2, with 2 to 89 times less penetration success than on susceptible wheat. This result is in line with previous works studying different resistances against *Z. tritici*. For instance, *Stb16q*-mediated full resistance mainly stops avirulent isolates during the penetration process, following pattern 1 [37]. Likewise, no evidence of successful penetration was observed for the IPO323 isolate when infecting *B. distachyon* [8]. The cytological study of eight interactions between *Triticum monococcum* and *Z. tritici* also highlighted an absence of symptoms and of sporulation when hyphae stopped during penetration, as our pattern 1 [15], suggesting that the resistance mediated by *T. monococcum* is also associated with stomata resistance. A direct correlation between the plant resistance efficiency and the fungus penetration success rate was recently reported for the wheat accession Runal infected with *Z. tritici* isolates carrying different isoforms of the avirulence gene *Avr3D1*, the less penetration success, the more resistance [25]. These observations, together with our results, strongly support the emerging idea that controlling stomatal penetration is a common crucial key for plant resistances to *Z. tritici* of all types [37, 63].

### Are they shared molecular and physiological mechanisms underlying plant resistance to *Z. tritici*?

So far, all the identified *Stb* genes encode receptor-like kinases (RLK) that recognize (for *Stb6* and *Stb9*) or likely recognize (for others) the pathogen through R/Avr gene-for-gene interactions [19, 20, 39, 40, 64]. Molecular and physiological studies of *Stb*-mediated resistances have highlighted similarities in the responses of *Stb6* and *Stb16q* to an avirulent isolate, in particular the induction of stomatal resistance [36, 37, 41]. The similarities between *Stb* proteins (RLKs), the probable similar pathogen recognition process (R/Avr gene-for-gene interactions), the physiological responses induced (stomatal resistance) and the stopping pattern of *Z. tritici* (cluster 1) suggest that *Stb*-mediated resistances may rely on similar mechanisms. These shared mechanisms could involve recognition of the fungus by the resistant plant during stomata penetration as avirulence gene are expressed at this early stage of infection [41] and the rapid induction of similar downstream responses that would ultimately lead to a major inhibition of fungal growth during stomatal penetration. One of the differences in these mechanisms may rely in the nature of its trigger, since the identified *Stb* genes encode for RLKs with extracellular domains of different nature [19, 39, 40].

Interestingly, NHR (except the one mediated by tobacco) and HSSR displayed stopping pattern of *Z. tritici* similar to *Stb*-mediated resistances (pattern 1). The similarity between strong resistances of different types questions the diversity of the underlying molecular and physiological mechanisms. On the one hand, a recognition process similar to *Stb*-mediated recognition process is conceivable for NHR and HSSR. While long known that plants recognize pathogens through R/Avr gene-for-gene interactions [65], it was also recently suggested that Avr participate to pathogen recognition during plants NHR [6, 66, 67]. Homologues of *Z. tritici* avirulence gene *Avr3D1* from *Zymoseptoria ardabiliae* and *Zymoseptoria pseudotritici* sharing 53 and 60% of protein sequence identity with *Avr3D1* were also recognized by resistant bread wheat cultivars as functional *Avr3D1 Z. tritici* proteins [25]. From this observation, one can speculate that *Stb* homologues from other species than bread wheat may recognize *Z. tritici* Avr and contribute to NHR and HSSR. Other responses might be common between resistant wheat and non-host plant species. For instance, ROS accumulation at the site of attempted stomatal penetration has been reported both during wheat *Stb6*-mediated resistance and during *B. distachyon* NHR [8, 34]. Taken together, these observations and our results, support an emerging conceptual model [63] in which all, or most, types of resistance that stop *Z. tritici* at the stomata rely on the same scheme, starting with an or a multiple R/Avr gene-for-gene recognition process and similar downstream resistance responses leading to a strong inhibition of fungal stomatal penetration.

Regarding quantitative resistances, no functional studies of downstream molecular and physiological mechanisms have been published. However, Meile et al. demonstrated that partial host resistance was observed with some haplotypes of *Z. tritici Avr3D1* likely recognized by *Stb7* [25]. Moreover, Langlands-Perry et al. suggested that the *G_07189* gene has a quantitative effect on Renan wheat accession resistance depending on the genetic background of the *Z. tritici* isolate. *G_07189* likely corresponds to *AvrStb20q* interacting with *Stb20q* [18, 68]. Together, these studies suggest that quantitative resistances can rely on R/Avr-gene-for-gene interactions as recognition process. Here, we demonstrated that quantitative resistances inhibited *Z. tritici* infection following pattern 2, being less effective in stopping stomatal penetration than pattern 1. Based on these results and with the assumption of a common resistance mechanism, we hypothesized that the difference observed between resistances rely in the efficiency of recognition by the plant and in its timing. According to this hypothesis, an earlier or stronger recognition (a stronger expression of R or Avr proteins and/or a stronger affinity between the R and Avr haplotypes) would lead to an earlier and/or stronger activation of downstream resistance responses, and consequently to an early inhibition of fungal infection (i.e. at the stomata), and hence to a stronger resistance. On the contrary, a later or weaker recognition of the fungus (a weaker expression of R or Avr proteins and/or a weaker affinity between the R and Avr haplotypes) would lead to a later activation of downstream resistance responses (i.e. in the apoplast), which has been shown to be less effective in stopping the fungus, and hence lead to a weaker resistance. The characterization of the molecular and physiological mechanisms underlying these various resistances to *Z. tritici*, their intensity and spatio-temporal deployment, is now essential to confirm or refute this model.

## Conclusions

Our results provide strong evidence that stomatal resistance is a common key process triggered by different types of resistance of wheat and non-host plants to *Z. tritici*. These results raise the questions of whether these different types of resistances rely on similar molecular mechanisms to control the fungal penetration process.

### Electronic supplementary material

Below is the link to the electronic supplementary material.

Supplementary Material 1

## Data Availability

The datasets used and/or analysed during the current study and the genetic resources are available from the corresponding author on reasonable request.
