# Supplementary figures and images for: Stomatal penetration: the cornerstone of plant resistance to the fungal pathogen Zymoseptoria tritici
Source: BMC Plant Biol. 2024 Aug 2;24:736. doi: 10.1186/s12870-024-05426-5 (PMC11295904; doi:10.1186/s12870-024-05426-5)

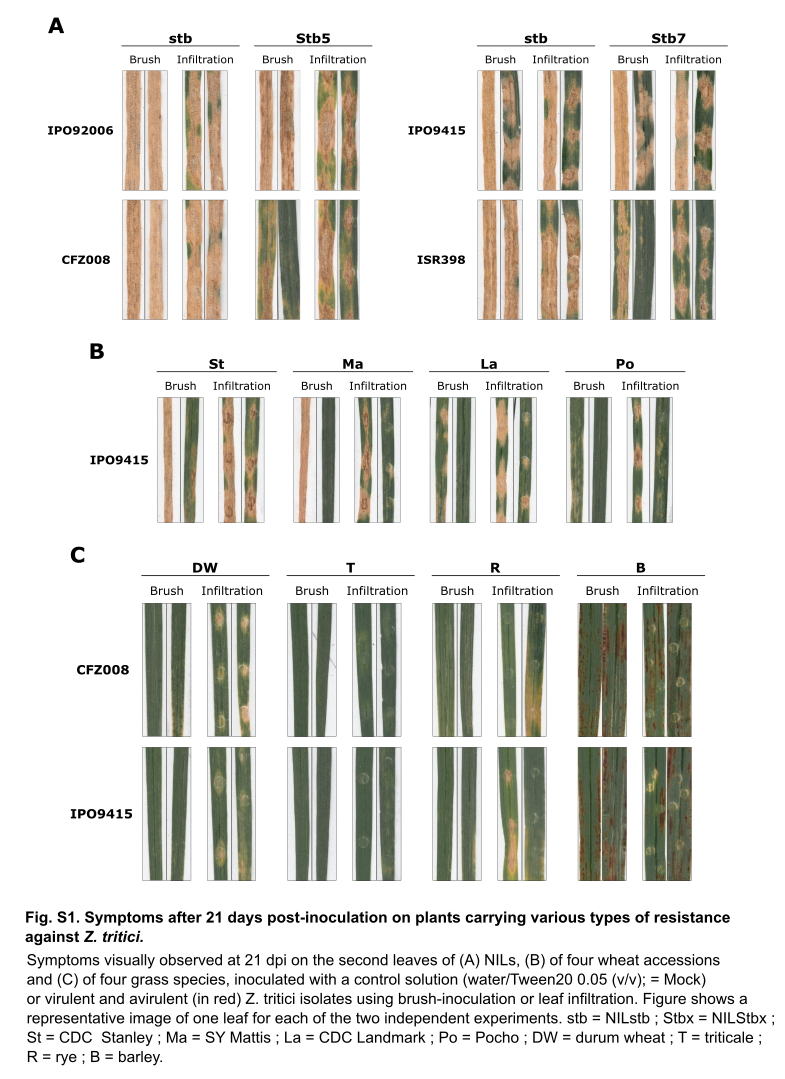

Supplement: Supplementary file 1 — Supplementary Material 1 [file 12870_2024_5426_MOESM1_ESM.tiff]
